# Primary Localization and Tumor Thickness as Prognostic Factors of Survival in Patients with Mucosal Melanoma

**DOI:** 10.1371/journal.pone.0112535

**Published:** 2014-11-10

**Authors:** Tarun Mehra, Gerd Grözinger, Steven Mann, Emmanuella Guenova, Rudolf Moos, Martin Röcken, Claus Detlef Claussen, Reinhard Dummer, Stephan Clasen, Aline Naumann, Claus Garbe

**Affiliations:** 1 Department of Dermatology, Eberhard-Karls-University, Tübingen, Germany; 2 Department of Diagnostic and Interventional Radiology, Eberhard-Karls-University, Tübingen, Germany; 3 Department of Dermatology, University Hospital of Zürich, Zürich, Switzerland; 4 Medical Directorate, UniversitätsSpital Zürich, Zürich, Switzerland; 5 Institute for Clinical Epidemiology and Applied Biometry, Eberhard-Karls-University, Tübingen, Germany; University of Connecticut Health Center, United States of America

## Abstract

**Background:**

Data on survival with mucosal melanoma and on prognostic factors of are scarce. It is still unclear if the disease course allows for mucosal melanoma to be treated as primary cutaneous melanoma or if differences in overall survival patterns require adapted therapeutic approaches. Furthermore, this investigation is the first to present 10-year survival rates for mucosal melanomas of different anatomical localizations.

**Methodology:**

116 cases from Sep 10 1984 until Feb 15 2011 retrieved from the Comprehensive Cancer Center and of the Central Register of the German Dermatologic Society databases in Tübingen were included in our analysis. We recorded anatomical location and tumor thickness, and estimated overall survival at 2, 5 and 10 years and the mean overall survival time. Survival times were analyzed with the Kaplan-Meier method. The log-rank test was used to compare survival times by localizations and by T-stages.

**Principal Findings:**

We found a median overall survival time of 80.9 months, with an overall 2-year survival of 71.7%, 5-year survival of 55.8% and 10-year survival of 38.3%. The 10-year survival rates for patients with T_1_, T_2_, T_3_ or T_4_ stage tumors were 100.0%, 77.9%, 66.3% and 10.6% respectively. 10-year survival of patients with melanomas of the vulva was 64.5% in comparison to 22.3% of patients with non-vulva mucosal melanomas.

**Conclusion:**

Survival times differed significantly between patients with melanomas of the vulva compared to the rest (p = 0.0006). It also depends on T-stage at the time of diagnosis (p<0.0001).

## Introduction

Primary mucosal melanoma is a rare neoplasm, accounting for approximately 1% of all melanomas [1]. Mucosal melanoma has been associated with a poorer prognosis than cutaneous melanoma [2] with 5-year disease-specific survival rates roughly a third of those seen in cutaneous melanoma (25.0% vs 80.8%) [1]. Adequate data allowing for the establishment of a reliable prognostic staging system are still sparse, although data from larger patient cohorts especially of those affected with mucosal melanoma of the head and neck have started to be published [3]. We here present data from 116 patients with mucosal melanoma from multiple anatomic sites with the aim of establishing prognostic markers as to help establish a classification system for primary mucosal melanomas. To our best knowledge, our sample of patients with primary mucosal melanoma is the largest published so far for mucosal melanomas of various anatomical locations and the only study publishing corresponding 10-year survival.

## Materials and Methods

### Patients

The Comprehensive Cancer Center database and the Central Register of the German dermatological Society both in Tübingen, were searched for cases of primary mucosal melanoma (malignant melanoma with a primary site being a mucosal epithelium of any anatomical region). The database of the Comprehensive Cancer Center yielded 48 cases and the database of the German dermatological Society 118 cases of mucosal melanoma, diagnosed or treated in Tübingen in a period from Sep 10 1984 until Feb 15 2011. After merging the two databases and excluding duplicates as well as cases with insufficient data, 116 cases were analyzed (Table 1). The observation period started on Sep 10 1984 and finished on Dec 22 2011. All cases analyzed had a clinically confirmed diagnosis. We recorded location, age, sex, tumor thickness, lymph node involvement, resection status, relapse and estimated overall survival at 2, 5 and 10 years and its mean.

**Table 1:** Characteristics of the study population.

Mucosal Melanoma							
	Total	Vulva	Vagina	Penis	Upper Airway	Conjunctiva	GI-Tract
**Number of Patients (%)**	**116 (100%)**	41 (35.3%)	6 (5.2%)	8 (6.9%)	36 (31.0%)	5 (4.3%)	20 (17.2%)
**Female Sex (%)**	**85 (73.3%)**	41 (100%)	6 (100%)	0 (0.0%)	23 (63.9%)	4 (80.0%)	11 (55.0%)
**Age (years)**							
Median	**66.5**	67.0	61.0	72.5	64.0	65.0	67.0
Range	**20–89**	20–83	46–68	32–80	41–89	43–80	45–80
**Thickness (n = 65, mm)**							
Median	2.9	2.0	6.5	2.0	5.0	0.7	7.0
Range	0.1–30.0	0.2–9.0	1.2–12.0	0.36–4.0	0.1–21.0	0.3–1.1	2.1–30.0
**Thickness** * **(n = 116, %)**							
TX	**35 (30.2%)**	5 (12.2%)	0	0	20 (55.6%)	2 (40.0%)	8 (40.0%)
Tis	**2 (1.7%)**	2 (4.9%)	0	0	0	0	0
T_1_	**10 (8.6%)**	6 (14.6%)	0	2 (25.0%)	1 (2.8%)	1 (20.0%)	0
T_2_	**18 (15.5%)**	11 (26.8%)	2 (33.3%)	2 (25.0%)	2 (5.6%)	1 (20.0%)	0
T_3_	**24 (20.7%)**	9 (22.0%)	0	4 (50.0%)	4 (11.1%)	1 (20.0%)	6 (30.0%)
T_4_	**27 (23.8%)**	8 (19.5%)	4 (66.7%)	0	9 (25.0%)	0	6 (30.0%)
**Lymph Node** * **(n = 116, %)**							
NX	**17 (14.7%)**	5 (12.2%)	0	0	8 (22.2%)	0	4 (20.0%)
N_0_	**81 (69.8%)**	30 (73.2%)	5 (83.3%)	6 (75.0%)	25 (69.4%)	5 (100%)	10 (50.0%)
N_1–3_	**18 (15.5%)**	6 (14.6%)	1 (16.7%)	2 (25.0%)	3 (8.3%)	0	6 (30.0%)
**Metastasis** * **(n = 116, %)**							
MX	**9 (7.8%)**	3 (7.3%)	0	0	5 (13.9%)	0	1 (5.0%)
M_0_	**102 (87.9%)**	38 (92.7%)	6 (100%)	8 (100%)	27 (75.0%)	5 (100%)	18 (90.0%)
M_1_	**5 (4.3%)**	0	0	0	4 (11.1%)	0	1 (5.0%)
**Resection (n = 102, %)**							
Total	**102**	40	6	8	26	3	19
RX	**44 (43.1%)**	11 (27.5%)	4 (66.7%)	2 (25.0%)	17 (65.4%)	1 (33.3%)	9 (47.4%)
R_0_	**44 (43.1%)**	25 (62.5%)	0	4 (50.0%)	7 (26.9%)	1 (33.3%)	7 (36.8%)
R_1–2_	**14 (13.7%)**	4 (10.0%)	2 (33.3%)	2 (25.0%)	2 (7.7%)	1 (33.3%)	3 (15.8%)
**Relapse**	**42 (36.1%)**	16 (39.0%)	4 (66.7%)	3 (37.5%)	12 (33.3%)	1 (20.0%)	6 (30.0%)
**(n = 116, %)**							
**Survival (n = 116)**							
2 years	**71.7%**	91.2%	53.3%	100%	59.0%	75.0%	47.4%
5 years	**55.8%**	78.6%	53.3%	83.3%	40.58%	75.0%	24.4%
10 years	**38.3%**	64.5%	53.3%	83.3%	21.31%	0.0%	0.0%
Mean (Months)	**93.3**	133.3	11.5	30.8	46.5	58.7	38.5
Median (Months)	**80.9**	185.2	N/A	N/A	48.7	71.5	19.5
Range (Months)	**0.7–296.4**	0.9–296.4	2.8–30.7	2.9–125.2	2.1–186.6	19.8–71.5	0.7–96.3
OD (n, %)	**49 (42.2%)**	11 (26.8%)	2 (33.3%)	1 (12.5%)	20 (55.6%)	2 (40.0%)	13 (65.0%)
CO (n, %)	**67 (57.8%)**	30 (73.2%)	4 (66.7%)	7 (87.5%)	16 (44.4%)	3 (60.0%)	7 (35.0%)

*Findings at diagnosis, classified according to AICC 7th edition (2009).

% indicates % of group, except Number of Patients, where % indicates % of total. Thickness: thickness of primary tumor. Resection: pathological description of resection status. Survival: overall survival. OD: observed deaths. CO: censored outcomes, N/A: not applicable (median could not be calculated, as over half the cases in this group were censored).

Clinical staging, follow-up and treatment were done at the Department of Dermatology, radiological staging and follow-up were done at the Department of Diagnostic and Interventional Radiology, both at the Eberhard-Karls-University of Tübingen, Germany.

Follow-up was carried out according to guidelines for cutaneous melanoma [4]. Maximum follow-up time was 297 months.

### Ethics

IRB approval was provided by the Ethics committee of the Medical Faculty of the University of Tübingen. The aforementioned IRB specifically approved this study. The study consisted in the retrospective analysis of already present clinical data of previously treated patients at our institution. No consent, written or oral, was obtained retrospectively. The aforementioned IRB specifically approved this consent procedure, as mentioned in the written IRB statement submitted as supplementary material together with the manuscript.

### Statistical analysis

The statistical analysis was done using JMP 10.0 for Mac [5] and R 3.1.0 using the survival-package [6] respectively. Survival time was defined as being the duration between the date of diagnosis and the date of death from any cause (overall survival) and was assessed with the Kaplan-Meier method [7]. The pointwise 95% confidence intervals from the estimated survival probability at 2-, 5- or 10-years are based on a log-log-transformation, described as log-transformation by Klein and Moeschberger [8]. The log-rank test was used to compare survival times by T-stage, using different subgroups of patients (all or N_0_M_0_) and by localization (vulva vs non-vulva). Assuming an overall significance level of α = 0.05, we adjusted for multiple testing (n = 3) by only considering results as “significant” if p<0.017 (Bonferroni correction).

Where applicable, findings were classified according to AICC 7th Edition, 2009.

## Results

### Patients and tumor localization

116 patients were included (Table 1). Of these, 85 (73.3% of total) were female. The median age at diagnosis was 66.5 years, ranging from 20 to 89 years. The anatomical sites of the primary were vulva (41 cases, 35.3% of total), vagina (6 cases, 5.2%), penis (8 cases, 6.9%), upper airway including nasal/paranasal sinuses as well as the oral cavity (36 cases, 31.0%) conjunctiva (5 cases, 4.3%) and the gastrointestinal tract (GI-tract) including anus (20 cases, 17.2%). The sojourn time of patients in the study ranged from 1 to 297 months; its median was 28.5 months with an interquartile range of 12.5 to 70.5 months.

### Tumor thickness

Of 116 patients, 65 had a documented tumor thickness of the primary at the time of diagnosis, the median tumor thickness was 2.9 mm and ranging from 0.1–30.0 mm.

Mucosal melanomas situated in the GI-tract had the highest median tumor thickness (7.0 mm), followed by tumors of the vagina (6.5 mm) and of the upper airway (5.0 mm). Melanomas of the conjunctiva had the lowest median tumor thickness (0.7 mm), followed by the penis and the vulva (both 2.0 mm).

### Staging at time of diagnosis

The tumor of two patients (1.7% of total) were classified as Tis, 10 as T_1_ (8.6%), 18 as T_2_ (15.5%), 24 as T_3_ (20.7%) and 27 as T_4_ (23.8%). The remaining 35 patients (30.2%) had an unknown tumor thickness or T-Stage at the time of diagnosis and were classified as TX. Both Tis melanomas were melanomas of the vulva. The site with the highest percentage of T_4_ - tumors was the vagina (4 from 6, 66.7%, the 2 others being classified as T_2_) followed by the GI-tract (6 of 20, 30.0%) and the upper airway (9 from 36, 25.0%). Primary mucosal melanomas of the upper airway had the highest percentage of undocumented primary tumor thickness at the time of diagnosis (55.6%, 20 of 36 cases), followed by primaries of the GI-tract (8 of 20 cases, 40.0%) and of the conjunctiva (2 of 5 cases, 40.0%).

At the time of diagnosis, 81 patients (69.8%) did not have an involvement of the regional lymph nodes whereas 18 patients (15.5%) did and 17 patients (14.7%) had an unknown nodal status. At the time of diagnosis, 5 cases of metastatic disease (M_1_, 4.3%) were reported, 102 patients did not have detectable metastases (M_0_, 89.7%) and 9 cases were of unknown metastatic state (MX, 7.8%).

Of the 116 patients, 102 were operated. Of these, 44 had an unknown resectional status (RX, 43.1%), 44 were classified as R_0_ (43.1%) and 14 as R_1_ (13.7%).

During follow- up 42 of 116 patients suffered a relapse (36.1%).

### Site of primary tumor and survival

**Figure 1:**
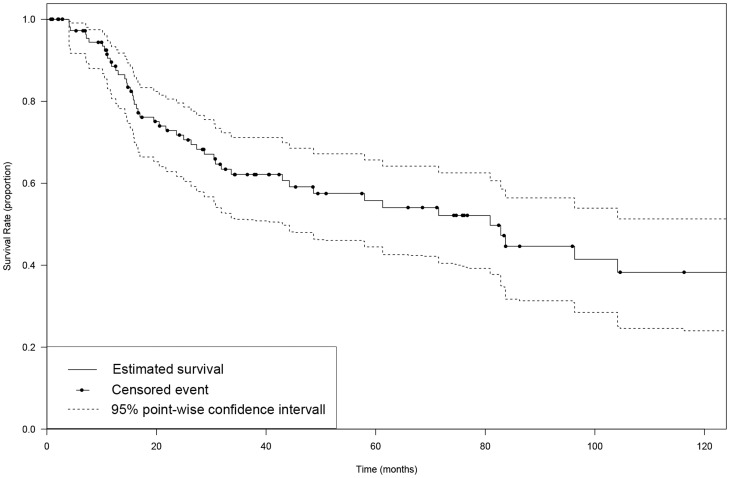
Overall 10-year survival of all cases of primary mucosal melanoma included in this study (n = 116).

**Figure 2:**
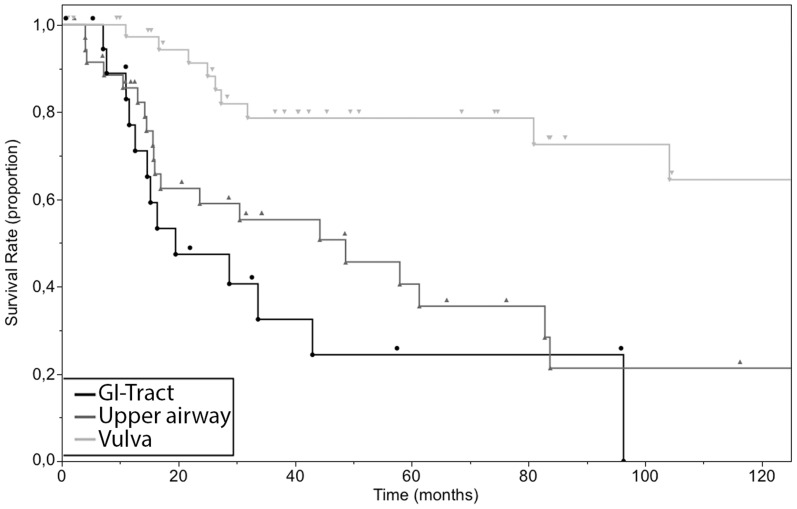
Overall 10-year survival of cases of primary mucosal melanoma according to the localization of the primary tumor. GI-Tract (n = 20), Upper airway (n = 36), Vulva (n = 41).

**Figure 3:**
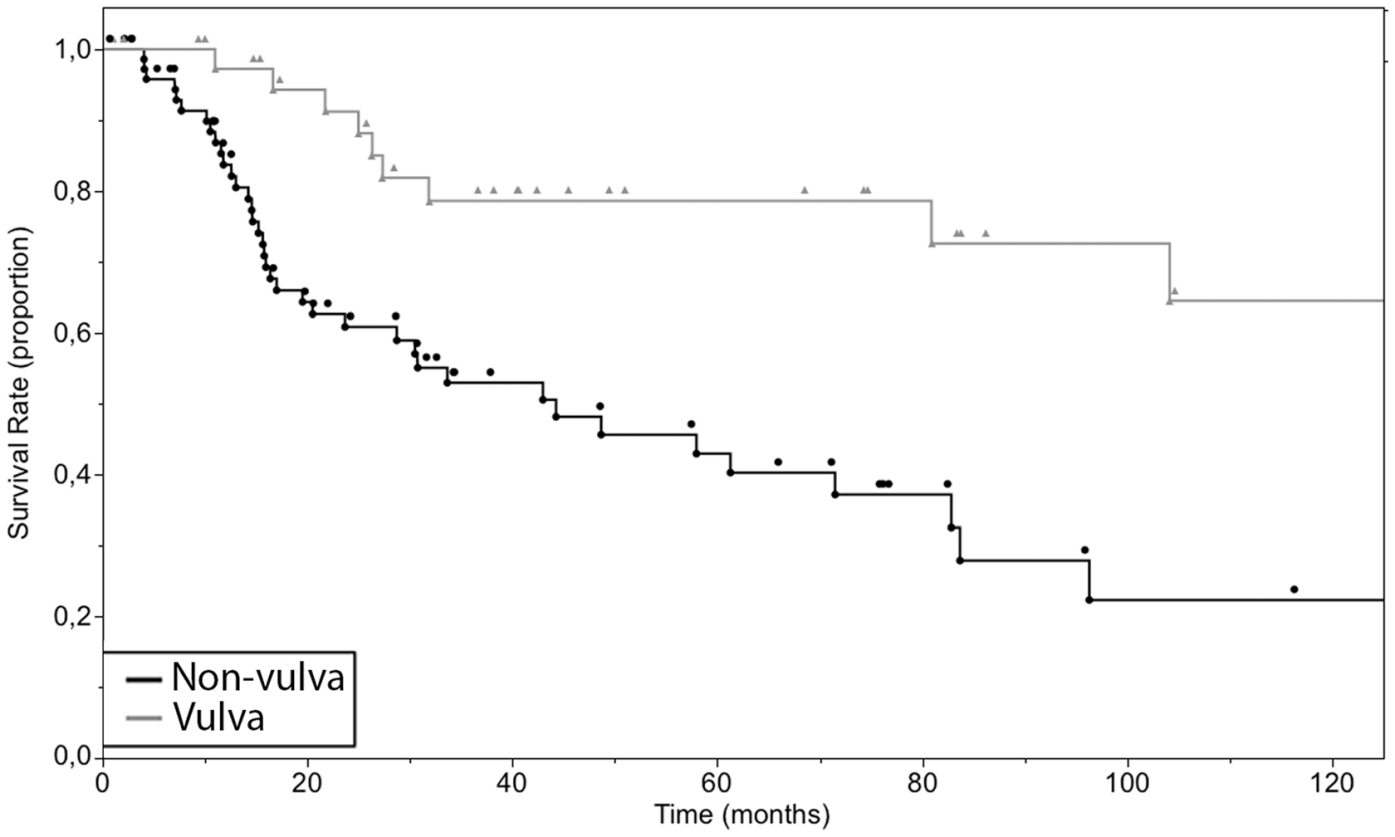
Overall 10-year survival of the 116 cases of primary mucosal melanoma grouped according to the localization of the primary tumor. Vulva (n = 41), Non-vulva (n = 75).

The overall survival times, combining patients in any anatomical site averaged 93.3 months, with a median overall survival of 80.9 months, ranging from 0.7 to 296.4 months (Figure 1, Figure S1). The group of patients with the lowest mean overall survival had a tumor at the vagina (11.5 months) followed by the penis (30.8 months) and GI-tract (38.5 months). The group of patients with the highest mean overall survival had a tumor at the vulva (133.3 months) followed by conjunctiva (58.7 months) and upper airway (46.5 months). The overall 2-year survival of patients with all sites of the primary was 71.7% (95% confidence interval (CI): 61.7% to 79.6%), 5-year survival was 55.8% (95% CI: 44.5% to 65.7%) and 10-year survival was 38.3% (95% CI: 24% to 51.3%). We also performed a survival analysis with the Kaplan-Meier method of the three cohorts with the highest number of cases (vulva, upper airway and GI-tract) (Figure 2). A clear distinction between the curve of the survival rate of patients with a primary of the vulva and the survival curve of the two other groups was observed. We did not test for significance due to overcrossing Kaplan-Meier curves of the survival rate of cases with primary tumors of the upper airway and of the GI-tract. As the data suggested a marked difference in survival times between vulva and non-vulva mucosal melanomas, we subsequently analyzed the survival of the cohort with a primary of the vulva (11 events in 41 cases) compared to the rest (38 events in 75 cases). 5 and 10-year overall survival were 78.6% (95% CI: 55.2% to 89.2%), 64.5% (95% CI: 37.6% to 81.4%) and 42.9% (95% CI: 29.2% to 55.9%), 22.3% (95% CI: 7.9% to 39.1%) for patients with a melanoma of the vulva and non-vulva respectively with corresponding median survival times of 185.2 months and 44.3 months (Figure 3). The survival times of patients with a melanoma of the vulva were significantly different from that of the rest (p = 0.0006).

### Impact of tumor thickness on survival

**Figure 4:**
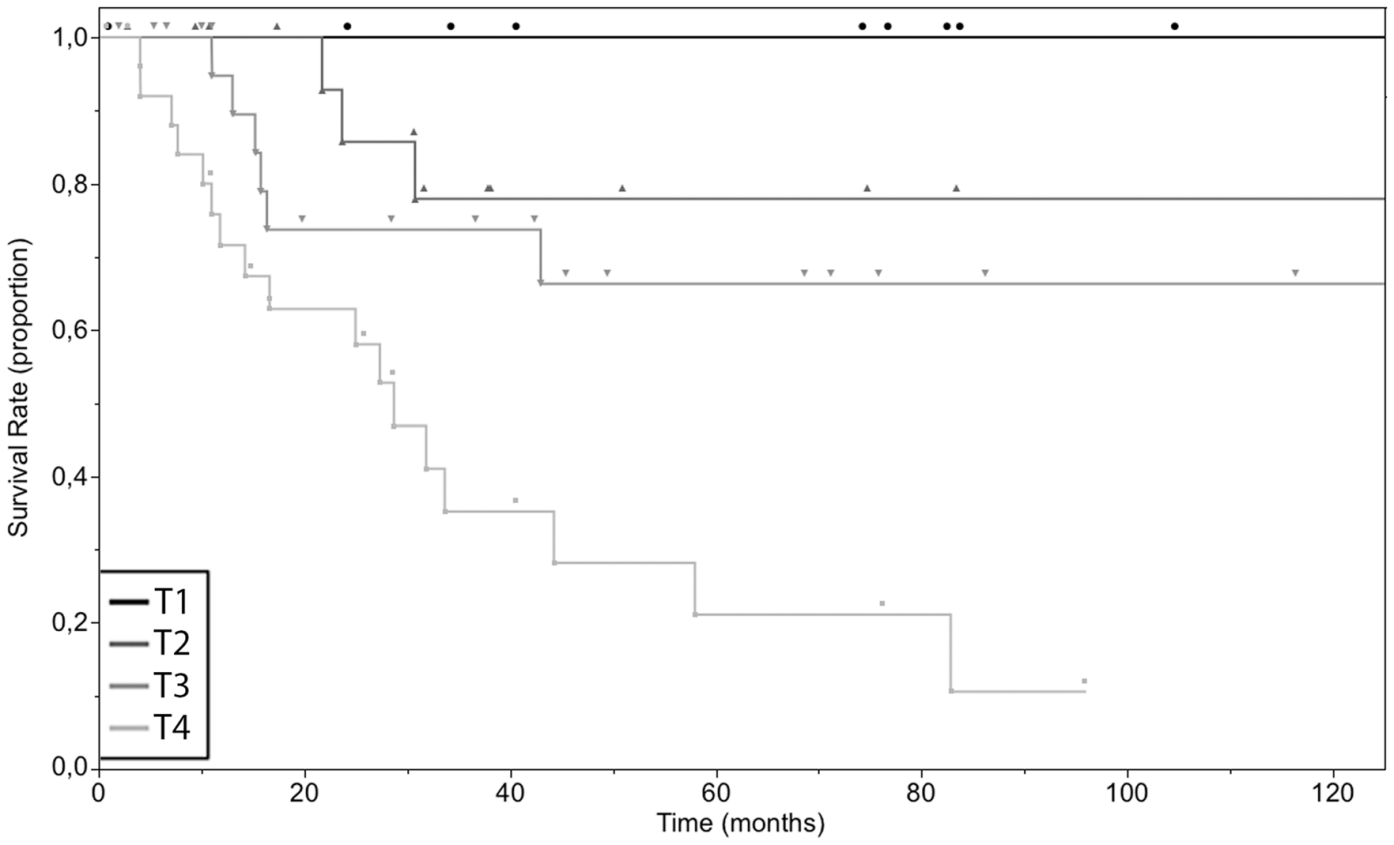
Overall 10-year survival of the 79 cases of primary mucosal melanoma classified as T_1_ (n = 10), T_2_ (n = 18), T_3_ (n = 24), T_4_ (n = 27), according to the tumor thickness of the primary at diagnosis. Tumors classified according to the AICC, 7th edition (2009).

All the patients with a primary tumor classified as Tis or T_1_ (n = 2 and n = 10 respectively) had a 10-year overall survival of 100% (lower limit of 95% CI: 100%). For patients with tumors classified as T_2_ (4 events in 18 cases), the 5- and 10-year survival were 77.9% (95% CI: 35.4% to 92.3% and 25.9% to 92.3% respectively). The 5- and 10-year survival rate of patients with T_3_-staged primaries (6 events in 24 cases) were both 66.3% (95% CI: 35.6% to 83.5% and 15.7% to 83.5% respectively). The 5-year survival rate of patients with T_4_-staged primaries (17 events in 27 cases) was 21.1% (95% CI: 5.8% to 42.7%). 10-year survival for patients with T_4_ tumors could not be estimated. The last living patient was censored after 96 months when the survival curve was at the level of 10.6%. Mean overall survival times were 118.3, 35.4 and 36.5 months for patients with T_2_, T_3_ or T_4_ tumor respectively (Table 2). Survival times of the groups were significantly different (p<0.0001) (Figure 4).

**Table 2:** Characteristics of the patient cohorts classified according to their T stage.

	Total	Tis	T_1_	T_2_	T_3_	T_4_
**Number of Patients (%)**	**81 (100.0%)**	2 (2.5%)	10 (12.3%)	18 (22.2%)	24 (29.6%)	27 (33.3%)
**Female Sex (%)**	64 (79.0%)	2 (2.5%)	8 (9.9%)	15 (18.5%)	18 (22.2%)	21 (25.9%)
**Age (years)**						
Median	64	66	50	64.5	70	70
Range	43–89	49–83	20–72	36–80	22–83	46–89
Lymph Node* (n = 81, %)						
NX	0	0	0	0	0	0
N_0_	67 (82.7%)	2 (100.0%)	10 (100.0%)	16 (88.9%)	18 (75.0%)	21 (77.8%)
N_1–3_	14 (17.3%)	0	0	2 (11.1%)	6 (25.0%)	6 (22.2%)
Metastasis* (n = 81, %)						
MX	0	0	0	0	0	0
M_0_	78 (96.3%)	2 (100.0%)	10 (100.0%)	18 (100.0%)	21 (87.5%)	27 (100.0%)
M_1_	3 (3.7%)	0	0	0	3 (12.5%)	0
**Resection (n = 81, %)**						
Total	77 (100.0%)	2 (100.0%)	10 (100.0%)	18 (100.0%)	21 (87.5%)	26 (96.3%)
RX	29 (37.7%)	0	5 (50.0%)	6 (33.3%)	5 (23.8%)	13 (50.0%)
R_0_	36 (46.8%)	2 (100.0%)	4 (40.0%)	11 (61.1%)	13 (61.9%)	6 (23.1%)
R_1–2_	12 (15.6%)	0	1 (10.0%)	155.6%)	3 (14.3%)	7 (26.9%)
**Relapse**	32 (39.5%)	0	3 (30.0%)	8 (44.4%)	9 (37.5%)	12 (44.4%)
**(n = 81, %)**						
**Survival (n = 81)**						
2 years	**76.6%**	100%	100%	85.7%	73.7%	62.9%
5 years	**59.0%**	100%	100%	77.9%	66.3%	21.1%
10 years	**55.0%**	100%	100%	77.9%	66.3%	N/A
Mean (Months)	**92.5**	N/A	N/A	118.3	35.4	36.5
Median (Months)	**144.6**	N/A	N/A	N/A	N/A	28.7
Range (Months)	**0.7–296.4**	1.0–245.6	0.9–296.4	2.9–186.6	2.0–137.6	0.7–95.9
OD (n, %)	**27 (33.3%)**	0 (0.0%)	0 (0.0%)	4 (22.2%)	6 (25.0%)	17 (63.0%)
CO (n, %)	**54 (66.7%)**	2 (100.0%)	10 (100.0%)	14 (77.8%)	18 (75.0%)	10 (37.0%)

T stage according to tumor thickness at diagnosis, classified according to AICC 7th edition (2009).

% indicates % of group, except Number of Patients, where % indicates % of total. Thickness: thickness of primary tumor. Resection: pathological description of resection status. Survival: overall survival. OD: observed deaths. CO: censored outcomes, N/A: not applicable (median could not be calculated, as over half the cases in this group were censored).

To exclude possible sources of bias, we restricted the following comparison of T_1_ (0 events in 10 cases), T_2_ (4 events in 16 cases), T_3_ (1 event in 15 cases) and T_4_ (13 events in 21 cases) to patients of subgroup N_0_M_0_ (no involvement of the regional lymph nodes and no distant metastasis). 5 and 10-year overall survival were 100.0% (lower limit of 95% CI: 100%) for T_1_N_0_M_0_, 76.2% (95% CI: 30.5% to 91.7% and 25.5% to 91.7% respectively) for T_2_N_0_M_0_, 88.9% (95% CI: 33.7% to 98.4% and 3% to 98.4% respectively) for T_3_N_0_M_0_. 10-year survival for patients with T_4_N_0_M_0_ tumors could not be estimated. The last living patient was censored after 96 months when the survival curve was at the level of 12.4%, the 5-year overall survival was 24.9% (95% CI: 6.6% to 49.1%). Mean overall survival times were 116.2, 43.0 and 38.3 months for T_2_N_0_M_0_, T_3_N_0_M_0_ and T_4_N_0_M_0_ cohorts (Table 3, Figure S2). The differences in survival time between groups was significant (p<0.0001).

**Table 3:** Characteristics of the patient cohorts classified according to their T stage, all N_0_M_0_.

Mucosal Melanoma						
	Total N_0_M_0_	TisN_0_M_0_	T_1_N_0_M_0_	T_2_N_0_M_0_	T_3_N_0_M_0_	T_4_N_0_M_0_
**Number of Patients (%)**	**64 (100.0%)**	2 (3.1%)	10 (15.6%)	16 (25.0%)	15 (23.4%)	21 (32.8%)
**Female Sex (%)**	**51 (79.7%)**	2 (3.9%)	8 (15.7%)	13 (25.5%)	12 (23.5%)	16 (31.4%)
**Age (years)**						
Median	**66.5**	66	50	64.5	73	68
Range	**20–89**	49–83	20–72	36–80	22–83	46–89
**Resection (n = 64, %)**						
Total	**62 (100.0%)**	2 (100.0%)	10 (100.0%)	16 (100.0%)	14 (100.0%)	20 (100.0%)
RX	**24 (38.7%)**	0	5 (50.0%)	5 (31.3%)	4 (28.6%)	10 (50.0%)
R_0_	**29 (46.8%)**	2 (100.0%)	4 (40.0%)	11 (68.8%)	8 (57.1%)	4 (20.0%)
R_1–2_	**9 (14.5%)**	0	1 (10.0%)	0	2 (14.3%)	6 (30.0%)
**Relapse**	21 (32.8%)	0	3 (30.0%)	6 (37.5%)	3 (20.0%)	9 (42.9%)
**(n = 64, %)**						
**Survival (n = 64)**						
2 years	**83.4%**	100%	100%	84.6%	100.0%	63.2%
5 years	**66.0%**	100%	100%	76.2%	88.9%	24.9%
10 years	**61.6%**	100%	100%	76.2%	88.9%	N/A
Mean (Months)	**101.5**	N/A	N/A	116.2	43.0	38.3
Median (Months)	**144.6**	N/A	N/A	N/A	N/A	27.3
Range (Months)	**0.7–296.4**	1.0–245.6	0.9–296.4	2.9–186.6	2.0–137.6	0.7–95.9
OD (n, %)	**27 (33.3%)**	0 (0.0%)	0 (0.0%)	4 (25.0%)	1(6.7.0%)	13 (61.9%)
CO (n, %)	**54 (66.7%)**	2 (100.0%)	10 (100.0%)	12 (75.0%)	14 (93.3%)	8 (38.1%)

T stage according to tumor thickness at diagnosis, classified according to AICC 7th edition (2009).

% indicates % of group, except Number of Patients, where % indicates % of total. Thickness: thickness of primary tumor. Resection: pathological description of resection status. Survival: overall survival. OD: observed deaths. CO: censored outcomes, N/A: not applicable (median could not be calculated, as over half the cases in this group were censored).

### Impact of lymph node involvement, metastasis and resectional status on survival

Out of 99 of 116 cases with a documented status of regional lymph node metastasis, 81 did not have (N_0_) and 18 had an involvement of the regional lymph nodes (N_1–3_). Patients staged as N_0_ had an overall 5-year survival of 56.2% and a 10-year survival of 42.9%. Those with a tumor burden in the regional lymph nodes had an overall 5-year survival rate of 44.5% (Figure S3). 10-year survival for patients with a tumor burden in the regional lymph nodes could not be estimated. The last living patient was censored after 75 months when the survival curve was at the level of 29.7%. A trend towards increased survival for N_0_ patients could be found.

Of 107 cases with an established status for distant metastasis, 102 did not have (M_0_) and 5 had distant metastasis (M_1_) at diagnosis. Patients staged as M_0_ had an overall 2-year survival rate of 73.9%, a 5-year survival of 58.1% and a 10-year survival of 41.4%. Those classified as M_1_ had an overall 2-year survival rate of 40%. At 5 years from diagnosis, no M_1_ patient remained in observation; the last patient died after 49 months (Figure S4). A trend towards increased survival for M_0_ patients could be found, although the very few cases of M_1_ patients (n = 5) has to be taken into consideration.

Of 58 cases with a documented tumor resection, 44 had tumor free borders (R_0_) and 14 had resection borders which were not (R_1–2_). R_0_-patients had an overall 5-year survival of 71.3% and a 10-year survival of 61.1%. R_1–2_-patients had an overall 2-year survival rate of 62.9% and a 5-year survival of 52.4% (Figure S5). 10-year survival for R_1–2_-patients could not be estimated. The last living patient was censored after 96 months when the survival curve was at the level of 38%.

**Figure 5:**
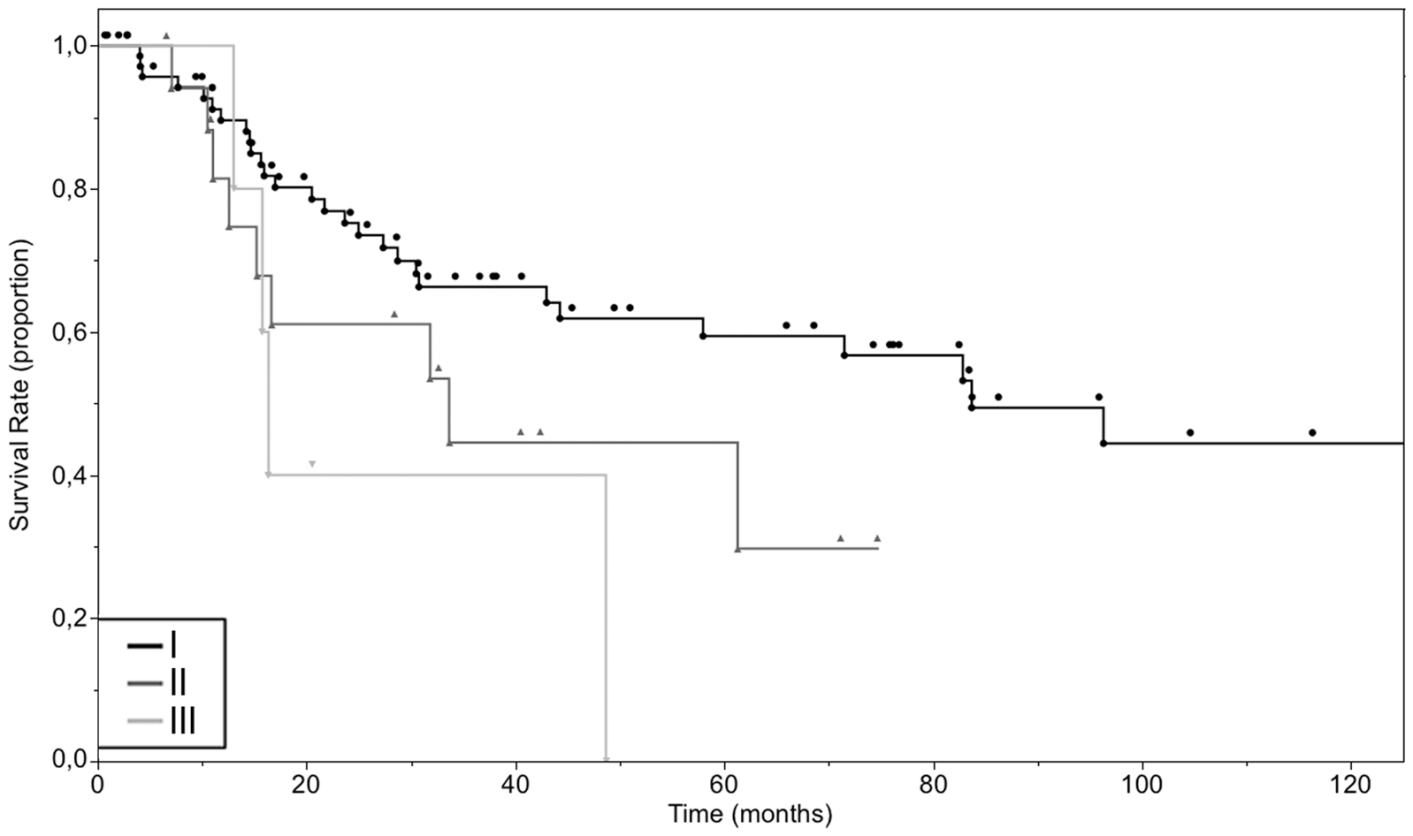
Overall 10-year survival of the 97 cases of primary mucosal melanoma which could be classified according to the Mucosal Melanoma Staging System [9]. Stage I: local disease (any T, N_0_, M_0_, n = 74), Stage II: regional lymph node involvement (any T, N_1–3_, M_0_, n = 18), Stage III: distant metastasis (any T, any N, M_1_, n = 5).

Then we retrospectively staged cases according to the Mucosal Melanoma Staging System published by Iversen et al. in 1980 [9], [10], which groups cases into regional disease (any T, N_0_, M_0_, Stage I, n = 74), involvement of the regional lymph nodes (any T, N_1–3_, M_0_, Stage II, n = 18) and distant metastasis (any T, any N, M_1_, Stage III, n = 5). Patients classified as Stage I had an overall 2-year survival of 75.2%, 5-year survival of 59.4% and 10-year survival of 44.4%. Corresponding survival for Stage II patients were 61.1% and 44.5%. 10-year survival for Stage II patients could not be estimated. The last living patient was censored after 75 months when the survival curve was at the level of 29.7%. 2-year survival for Stage III patients was 40%; at 5 years from diagnosis, no Stage III patient remained in observation; the last patient died after 49 months (Figure 5).

The trace of the Kaplan-Meier curve showed a trend towards a lower survival for the cohort of cases with extensive disease (Stage I) in comparison towards the cohort with regional disease (Stage II/III) (Figure S6).

## Discussion

Published 5-year survival for mucosal melanoma varies between 17 and 40% [11]–[14]. We observed an overall 5-year survival of 55.8%. Our survival rates were higher for patients of all anatomic regions except for those with a primary in the GI-tract [11]. Especially striking is our cohort of melanomas of the vulva and the penis, which have survival rates which approach those seen in cutaneous melanoma [15]. We report considerably higher overall survival rates for patients with mucosal melanoma than those reported by Kim et al. who found a 2-year survival rate of 59.7% and a 5-year survival rate of 31.9% [11]. The distribution of the anatomical sites was similar to our cohort, reporting a generally lower 5-year survival rate per group. Nonetheless, the sites of occurrence of the primaries were differently grouped, as our research combined anorectum and GI to GI-tract and nasal, oral and maxillary sinus to upper airway, but split genitourinary into penis, vulva and vagina. In accordance with the literature, our findings confirm the lower survival rates of mucosal melanoma [14]. Indeed, 5-year and 10-year survival for patients with cutaneous melanoma are approximately 80% and 70–80% respectively [14], [15].

It seems that independently of lymph node involvement and distant metastasis, survival times between T-stage groups are different. However, survival of mucosal melanoma patients in comparison to similar cohorts of cutaneous melanoma does not appear to be markedly different in the absence of lymph node involvement or metastasis, with the exception of mucosal melanomas with a high tumor thickness (>4.0 mm or T4).

The 5-year survival rates for patients with regional metastatic disease of mucosal melanoma seem to be poorer than those of cutaneous melanoma (stage II in our classification, 44.5% vs. 78%, 59% and 40% for IIIA, IIIB, IIIC respectively) [16].

The one censored case considered apart, the four other cases with a stage M_1_ at diagnosis died before reaching the 5-year time-point; the last patient died after 49 months (vs. 5-year survival of 10–20% for M_1b_ and M_1c_ combined) [16]. Therefore it seems that the poorer prognosis of mucosal melanoma can be attributed to a more aggressive metastatic behavior, which coincides with the author's clinical observations.

We chose not to stage our cases with the TNM staging system, although it would have made results more comparable, as the cohorts were too small for the different TNM sub-splits.

One astonishing result was the significantly superior survival of patients with a mucosal melanoma of the vulva in comparison to the rest. Nonetheless, in this region the epithelium changes from mucosal to squamous, so it is arguable how many of these melanomas were actually mucosal in sensu stricto. However, the significantly superior survival rate could be influenced by differences in the tumor thickness: although the percentage of T_3_ and T_4_ stage primaries between the two groups was similar (T_3_: 22.0% vs. 20.0% and T_4_: 19.5% vs. 25.3% for vulva vs. non-vulva), the relative amount of Tis, T_1_ and T_2_ stage tumors was higher in the primaries localized at the vulva (4.9%, 14.6% and 26.8% for vulva, 0.0%, 5.3% and 9.3% for non-vulva) with a markedly higher proportion of TX tumors for non-vulval localizations (vulva: 12.2% vs. non-vulva: 40.0%). Anyhow, survival rates were similar to those seen in cutaneous melanoma [15]. We therefore would welcome a discussion on whether vulval melanoma should be considered mucosal or if it should be classified as a cutaneous malignancy.

In summary, we strongly advocate including tumor thickness and a distinction between vulval and non-vulval mucosal melanoma when designing a staging system if it is to have a prognostic value. We also advocate considering the establishment of separate therapeutic regimens for mucosal melanoma due to a more aggressive systemic disease. We further suggest considering melanoma of the vulva to be classified as a primary cutaneous neoplasm. The importance of tumor thickness in N_0_M_0_ patients, resectional status, lymph node affection and disseminated disease has to be validated by studies with larger cohorts.

The overall survival time of patients with mucosal melanoma depends on multiple factors. Using Kaplan-Meier method, we only analyzed univariate influences on the overall survival times. To cope with the multivariate influence, a Cox regression analysis would be necessary. Unfortunately, in our investigation the sample size of patients with complete information and the number of events recorded were too small to build a Cox regression model. Instead, we compared the patients with different T-stages in a subgroup of patient with N_0_M_0_ (no involvement of the regional lymph nodes and no distant metastasis).

## Conclusions

We are the first to present 10-year survival rates for patients with mucosal melanomas of different anatomical localizations and show that the anatomical localization of the primary mucosal melanoma (vulva vs. non-vulva) is a significant prognostic factor (p = 0.0006). We confirm the role of tumor thickness as a prognostic marker; the survival time of patients with mucosal melanoma depends on the primary's T-stage at the time of diagnosis (p<0.0001).

## Supporting Information

Figure S1 **Overall survival of all included cases of primary mucosal melanoma (n = 116).** The longest observation period per case amounted up to 300 months (25 years).
(TIF)

Figure S2 **Overall 10-year survival of cases of primary mucosal melanoma with local disease (T_1–4_, N_0_, M_0_, n = 62), grouped according to their tumor thickness at the time of diagnosis.** T_1_N_0_M_0_ (n = 10), T_2_N_0_M_0_ (n = 16), T_3_N_0_M_0_ (n = 15), T_4_N_0_M_0_ (n = 21).
(TIF)

Figure S3 **Overall 10-year survival of cases of primary mucosal melanoma grouped according to their status of lymph node involvement at the time of diagnosis.** N_0_ (n = 81), N_1–3_ (n = 18).
(TIF)

Figure S4 **Overall 10-year survival of cases of primary mucosal melanoma grouped according to their status of distant metastasis at the time of diagnosis.** M_0_ (n = 102), M_1_ (n = 5).
(TIF)

Figure S5 **Overall 10-year survival of cases of primary mucosal melanoma grouped according to their resectional status at the time of diagnosis.** R_0_ (n = 44), R_1–2_ (n = 14).
(TIF)

Figure S6 **Overall 10-year survival of cases of primary mucosal melanoma grouped to cases with local disease (T stages T_1–4_, N_0_, M_0_, n = 62) and cases with systemic disease at the time of diagnosis (all T stages, N_1–3_ and/or M_1_, n = 23).**
(TIF)
