# Supplementary figures and images for: Primary Localization and Tumor Thickness as Prognostic Factors of Survival in Patients with Mucosal Melanoma
Source: PLoS One. 2014 Nov 10;9(11):e112535. doi: 10.1371/journal.pone.0112535 (PMC4226547; doi:10.1371/journal.pone.0112535)

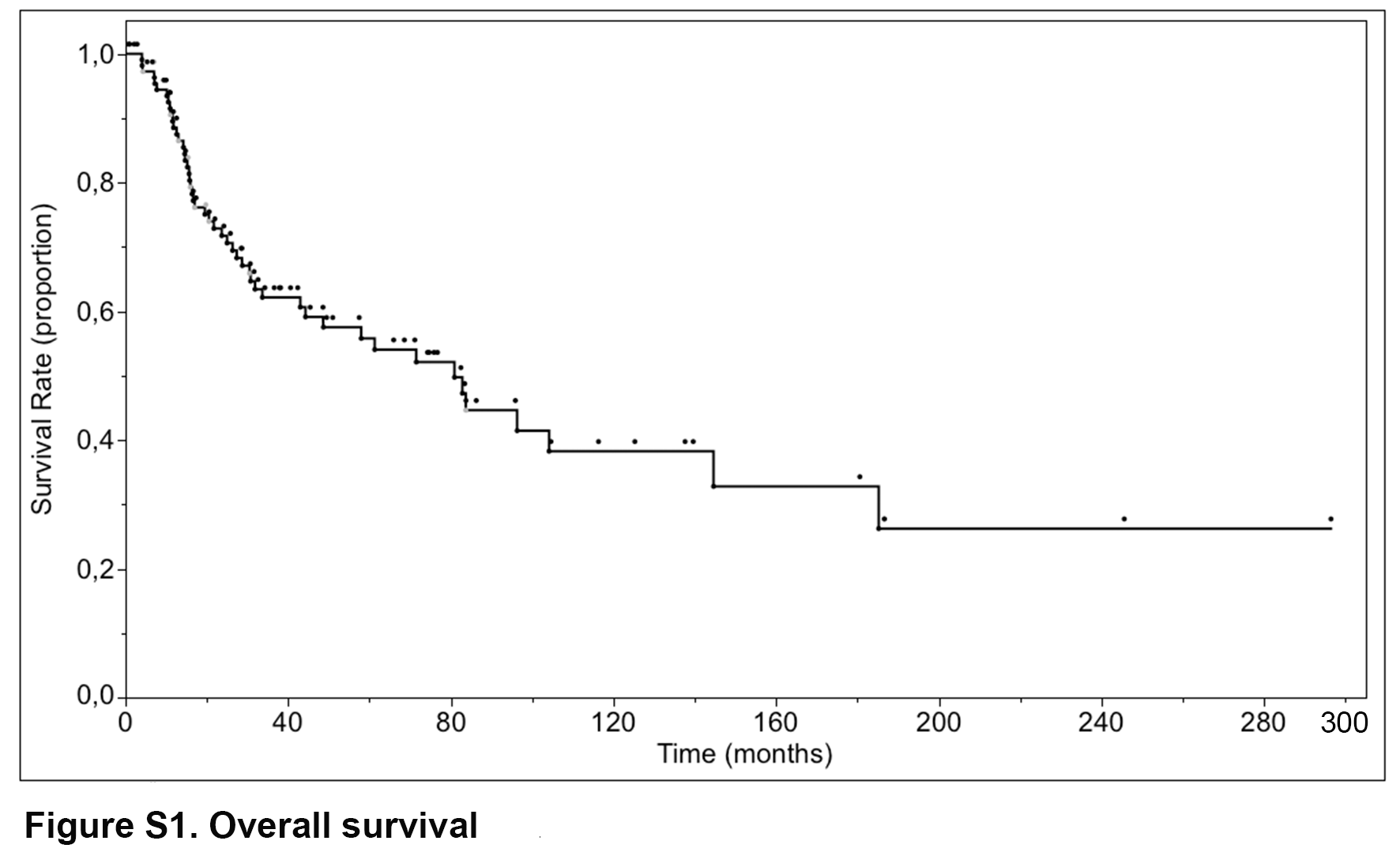

Supplement: Figure S1 — Overall survival of all included cases of primary mucosal melanoma (n = 116). The longest observation period per case amounted up to 300 months (25 years). (TIF) [file pone.0112535.s001.tif]

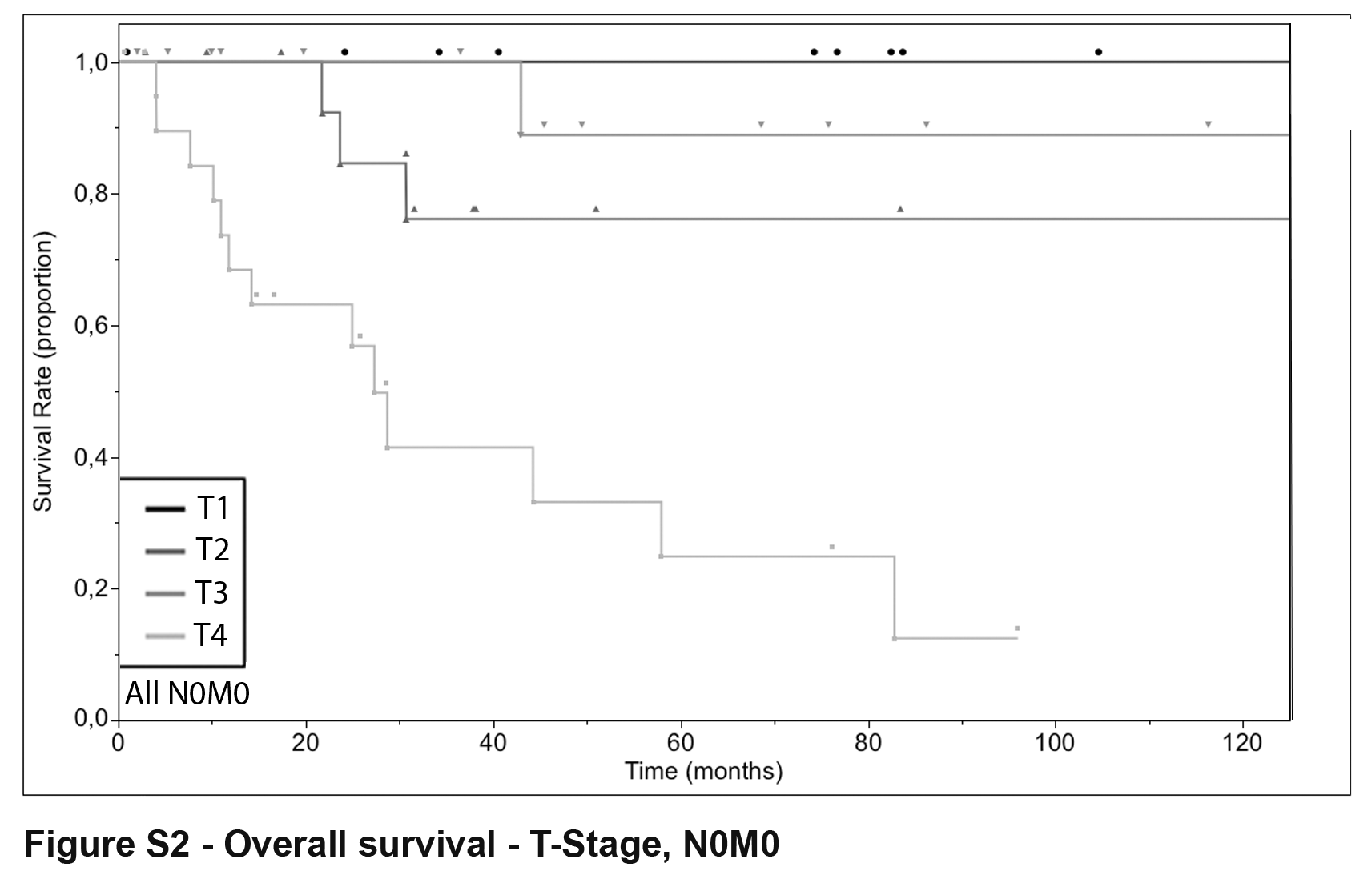

Supplement: Figure S2 — Overall 10-year survival of cases of primary mucosal melanoma with local disease (T1–4, N0, M0, n = 62), grouped according to their tumor thickness at the time of diagnosis. T1N0M0 (n = 10), T2N0M0 (n = 16), T3N0M0 (n = 15), T4N0M0 (n = 21). (TIF) [file pone.0112535.s002.tif]

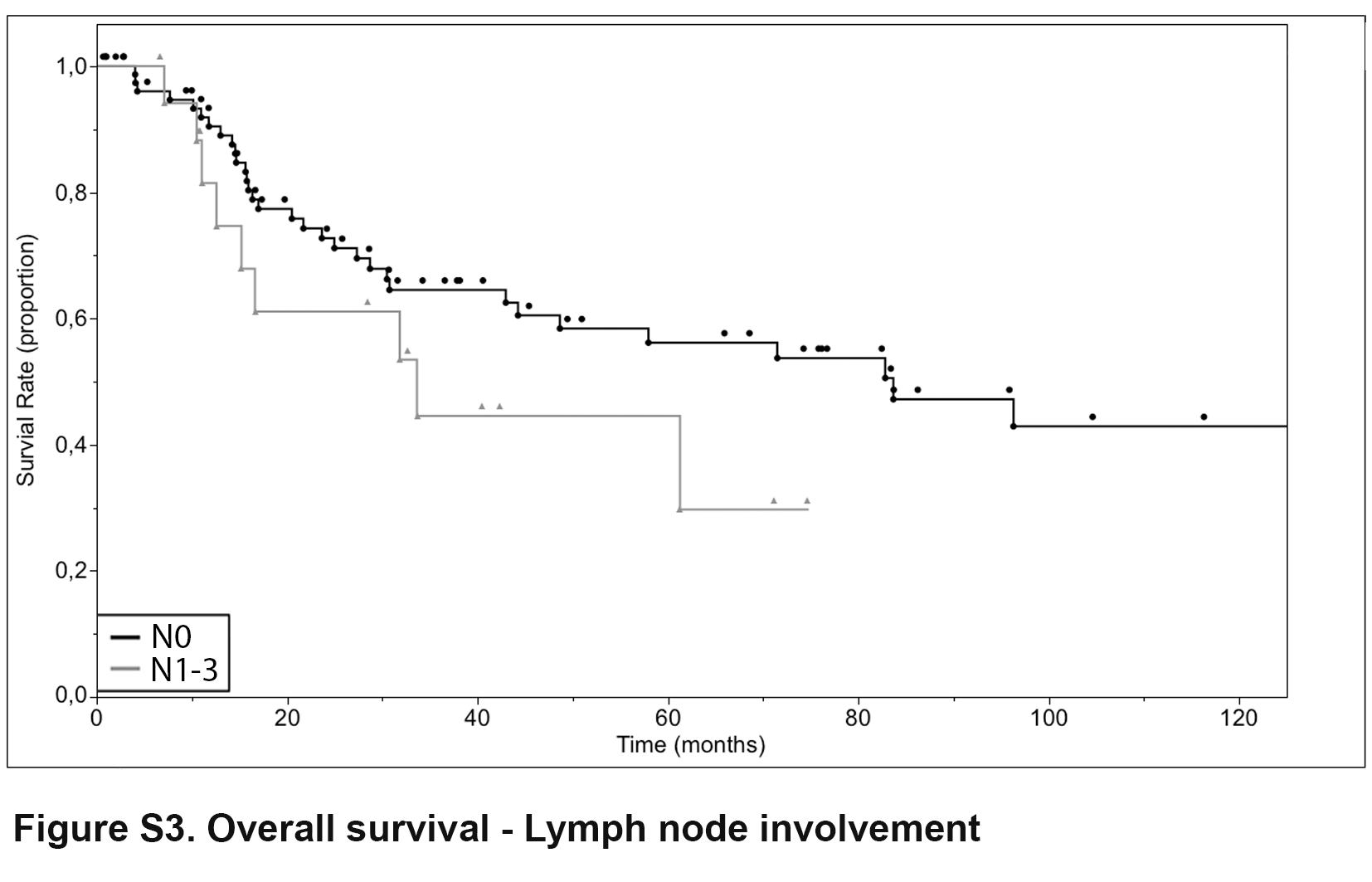

Supplement: Figure S3 — Overall 10-year survival of cases of primary mucosal melanoma grouped according to their status of lymph node involvement at the time of diagnosis. N0 (n = 81), N1–3 (n = 18). (TIF) [file pone.0112535.s003.tif]

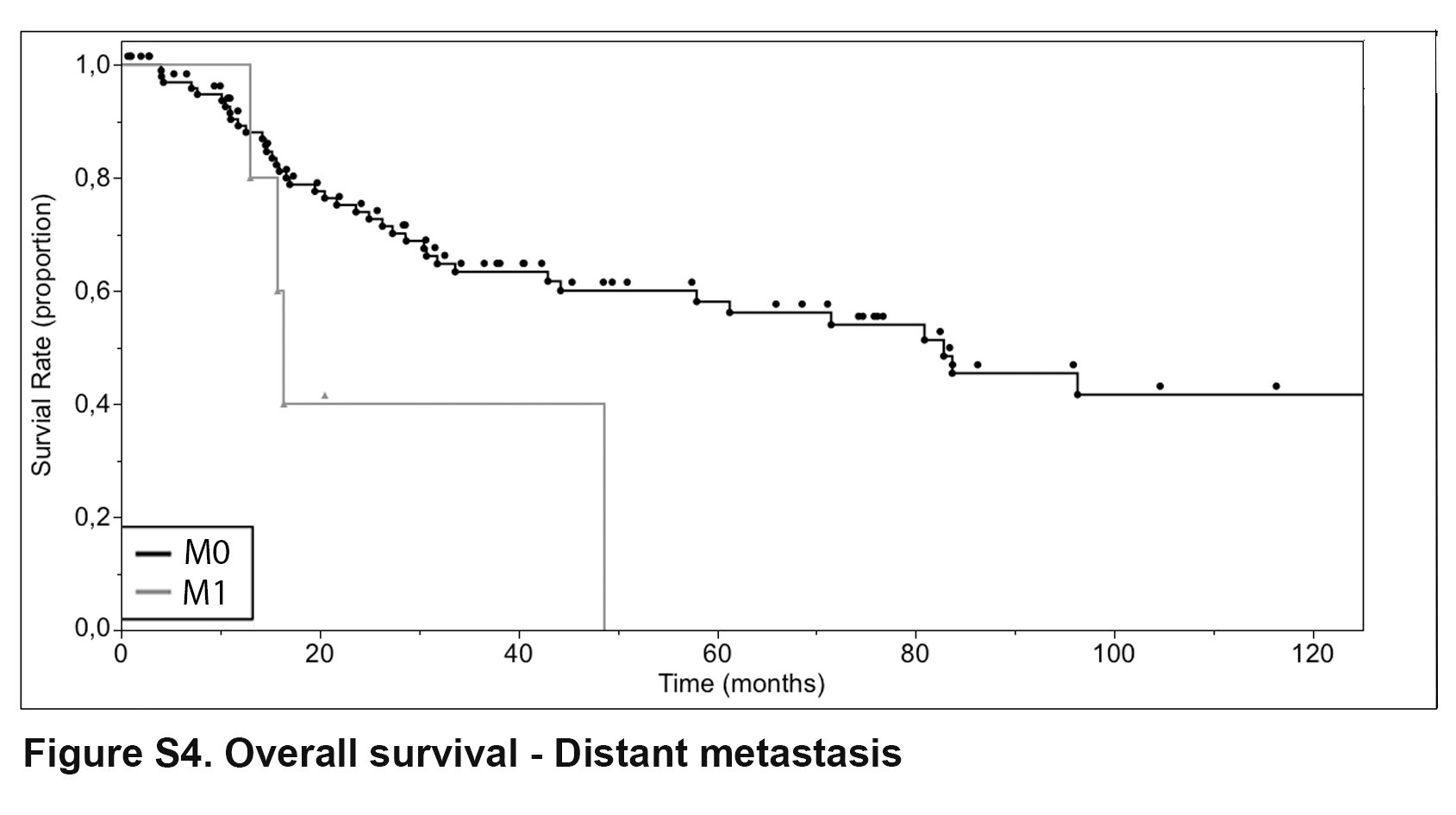

Supplement: Figure S4 — Overall 10-year survival of cases of primary mucosal melanoma grouped according to their status of distant metastasis at the time of diagnosis. M0 (n = 102), M1 (n = 5). (TIF) [file pone.0112535.s004.tif]

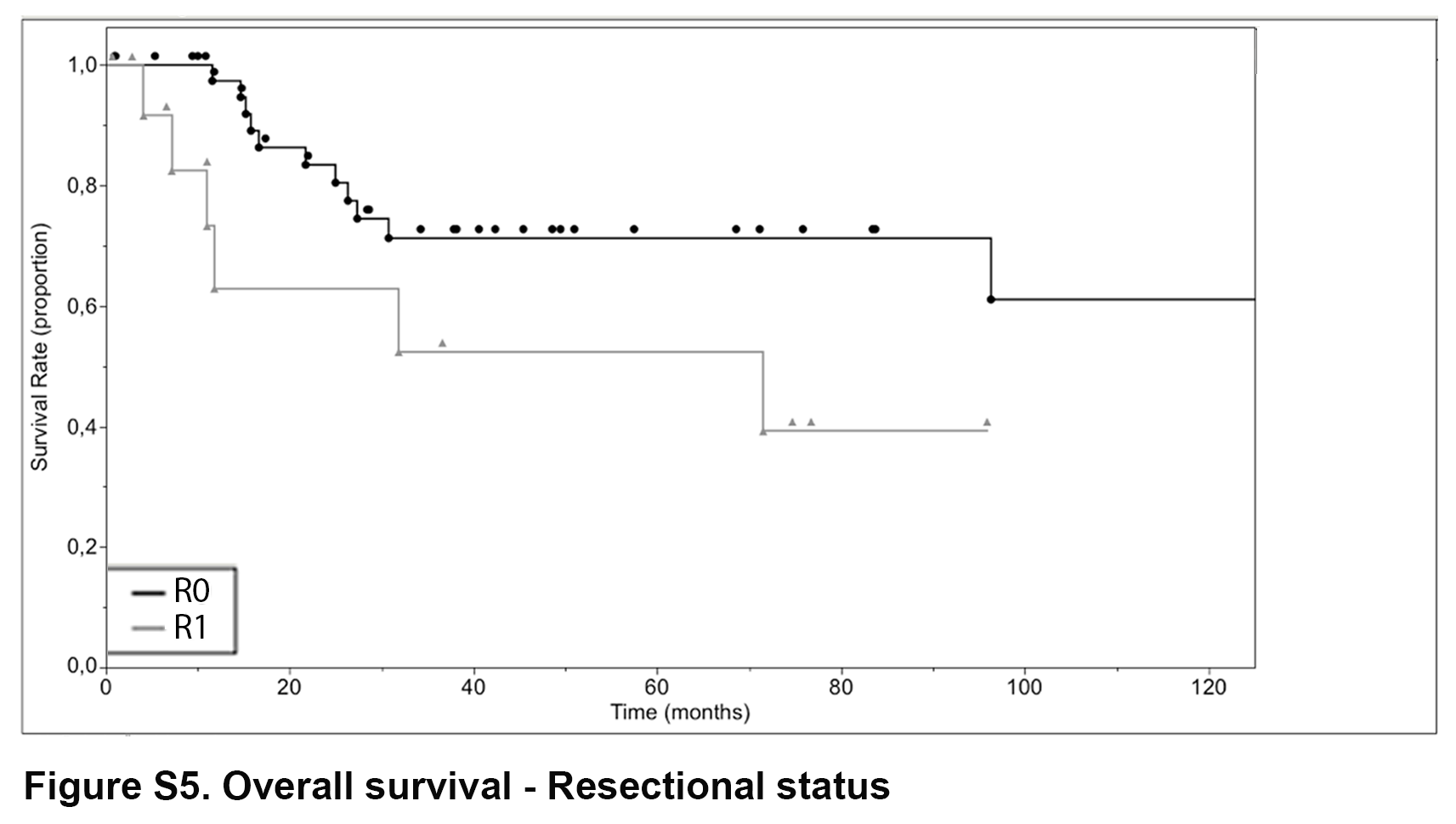

Supplement: Figure S5 — Overall 10-year survival of cases of primary mucosal melanoma grouped according to their resectional status at the time of diagnosis. R0 (n = 44), R1–2 (n = 14). (TIF) [file pone.0112535.s005.tif]

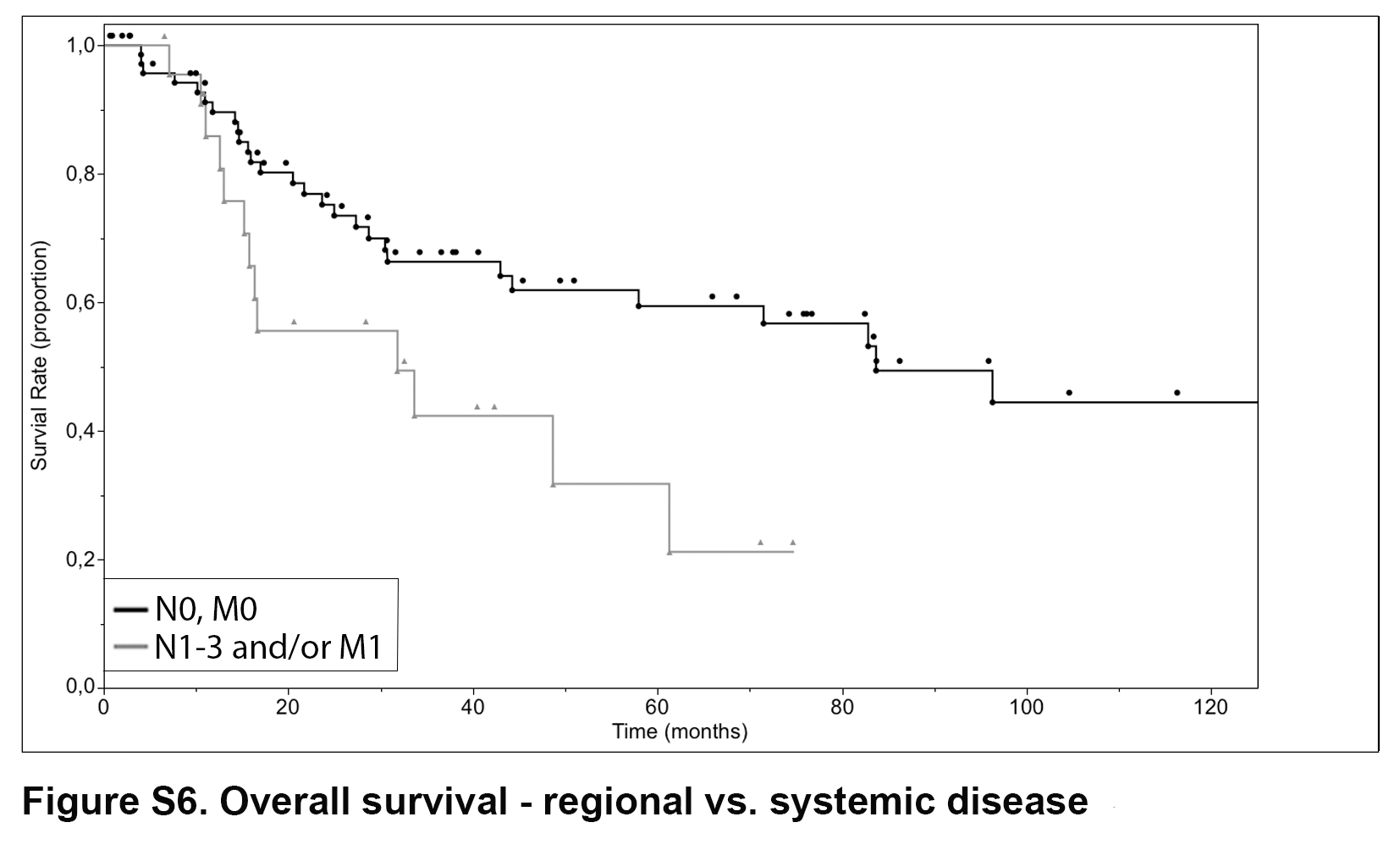

Supplement: Figure S6 — Overall 10-year survival of cases of primary mucosal melanoma grouped to cases with local disease (T stages T1–4, N0, M0, n = 62) and cases with systemic disease at the time of diagnosis (all T stages, N1–3 and/or M1, n = 23). (TIF) [file pone.0112535.s006.tif]
